# Genetic and environmental factors driving congenital solitary functioning kidney

**DOI:** 10.1093/ndt/gfad202

**Published:** 2023-09-20

**Authors:** Sander Groen in ‘t Woud, Marleen M H J van Gelder, Iris A L M van Rooij, Wout F J Feitz, Nel Roeleveld, Michiel F Schreuder, Loes F M van der Zanden, for the SOFIA study group, J A E van Wijk, R Westland, K Y Renkema, M R Lilien, M G Keijzer-Veen, F J Kloosterman, M G Steffens, V Gracchi, B Zegers, P E Jira, H van der Deure, R W G van Rooij, E Wijnands-van den Berg, M Breukels, S M H B de Pont, E Harnisch, C M L van Dael, D Creemers, R de Moor, A Y Konijnenberg, E Knots, E C van der Kuur, M J Jacobs, M Koppejan-Stapel, A Pijning, E Dorresteijn, R W J Leunissen, R Rijlaarsdam, R del Canho, B Semmekrot, A Dings-Lammertink, I J M Nijhuis, M J van Ledden-Klok, L M van den Broek, C Meine Jansen, M C G Beeren, H E Blokland-Loggers, C Dorrepaal, L J W M Pierik, A L Tanja

**Affiliations:** Radboud University Medical Center, Department for Health Evidence, Nijmegen, The Netherlands; Radboudumc Amalia Children's Hospital, Department of Paediatric Nephrology, Nijmegen, The Netherlands; Radboud University Medical Center, Department for Health Evidence, Nijmegen, The Netherlands; Radboud University Medical Center, Department for Health Evidence, Nijmegen, The Netherlands; Radboudumc Amalia Children's Hospital, Division of Pediatric Urology, Department of Urology, Nijmegen, The Netherlands; Radboud University Medical Center, Department for Health Evidence, Nijmegen, The Netherlands; Radboudumc Amalia Children's Hospital, Department of Paediatric Nephrology, Nijmegen, The Netherlands; Radboud University Medical Center, Department for Health Evidence, Nijmegen, The Netherlands

**Keywords:** aetiology, CAKUT, congenital anomaly, gene–environment interaction, solitary functioning kidney

## Abstract

**Background:**

Congenital solitary functioning kidney (CSFK) is an anomaly predisposing to hypertension, albuminuria and chronic kidney disease. Its aetiology is complex and includes genetic and environmental factors. The role of gene–environment interactions (G×E), although relevant for other congenital anomalies, has not yet been investigated. Therefore, we performed a genome-wide G×E analysis with six preselected environmental factors to explore the role of these interactions in the aetiology of CSFK.

**Methods:**

In the AGORA (Aetiologic research into Genetic and Occupational/environmental Risk factors for Anomalies in children) data- and biobank, genome-wide single-nucleotide variant (SNV) data and questionnaire data on prenatal exposure to environmental risk factors were available for 381 CSFK patients and 598 healthy controls. Using a two-step strategy, we first selected independent significant SNVs associated with one of the six environmental risk factors. These SNVs were subsequently tested in G×E analyses using logistic regression models, with Bonferroni-corrected *P*-value thresholds based on the number of SNVs selected in step one.

**Results:**

In step one, 7–40 SNVs were selected per environmental factor, of which only rs3098698 reached statistical significance (*P* = .0016, Bonferroni-corrected threshold 0.0045) for interaction in step two. The interaction between maternal overweight and this SNV, which results in lower expression of the Arylsulfatase B (*ARSB*) gene, could be explained by lower insulin receptor activity in children heterozygous for rs3098698. Eight other G×E interactions had a *P*-value <.05, of which two were biologically plausible and warrant further study.

**Conclusions:**

Interactions between genetic and environmental factors may contribute to the aetiology of CSFK. To better determine their role, large studies combining data on genetic and environmental risk factors are warranted.

## Graphical Abstract

**Graphical Abstract:**
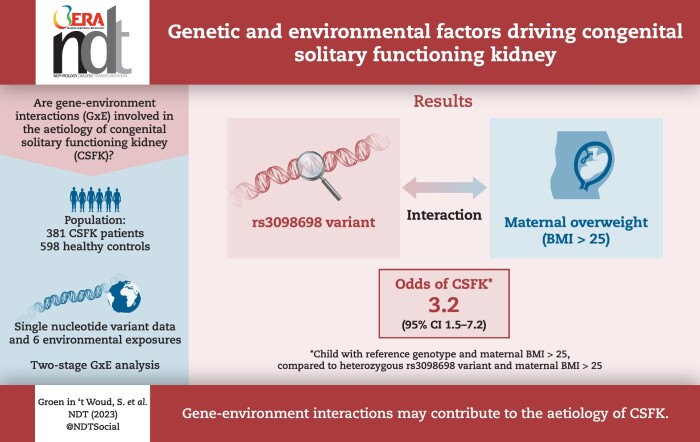


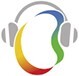
 Watch the video of this contribution at https://academic.oup.com/ndt/pages/author_videos

KEY LEARNING POINTS **What was known:**

- Congenital solitary functioning kidney (CSFK) is a congenital kidney anomaly predisposing to hypertension, albuminuria and chronic kidney disease.
- Genetic and environmental factors play a role in the aetiology of CSFK, but individually are insufficient to explain the relatively frequent occurrence of CSFK.
- For other congenital anomalies, research shows that the effects of exposure to environmental risk factors may lead to different effects based on the underlying genotype (gene–environment interaction).

**This study adds:**

- An interaction between the rs3098698 variant located in an intron of the Arylsulfatase B (*ARSB*) gene and maternal overweight or obesity was Bonferroni-corrected statistically significant, which is supported by *in silico* evaluation of the consequences of this variant.
- Eight other variants also reached a *P*-value for interaction <.05, but were not statistically significant after Bonferroni correction, indicating that more relevant interactions may be found in studies with additional power.

**Potential impact:**

- Gene–environment interactions may be relevant for CSFK and other congenital anomalies of the kidney and urinary tract (CAKUT), and should be included in future studies investigating CAKUT aetiology.
- As a combination of data on clinical, genetic and environmental risk factors is needed for comprehensive studies on the aetiology of CAKUT, studies including large numbers of patients for whom these data are available are warranted for adequate power.

## INTRODUCTION

A congenital solitary functioning kidney (CSFK) is a congenital anomaly which occurs in approximately 1:1500 children and results in hypertension, albuminuria and chronic kidney disease in up to 80% of patients by 18 years of age [1, 2]. Moreover, early signs of kidney injury, such as high blood pressure, can increase the risk of cardiovascular disease later in life [3]. The most important causes of CSFK are unilateral kidney agenesis, multicystic kidney dysplasia (MCDK) and kidney hypo-/dysplasia, which all fall within the spectrum of congenital anomalies of the kidney and urinary tract (CAKUT). Anomalies within the CAKUT spectrum are thought to share part of their aetiology because of the occurrence of different CAKUT phenotypes in family members with the same mutation [4]. Genetic, environmental and epigenetic factors may all be involved in CAKUT [5].

More than 150 monogenic causes for CAKUT have been reported [6]. The majority is associated with syndromic CAKUT, whereas only 23 genes are known to cause isolated CAKUT [7]. For CSFK, the diagnostic yield in studies aimed at identifying monogenic causes is usually low (7%–11%), suggesting that CSFK may have multiple causes [8–10]. Copy number variants (CNVs) contribute to the aetiology of CSFK, with 14%–17% of patients harbouring a rare CNV [11, 12]. In addition, common variants are likely to play a role, and we recently identified two candidate loci in a genome-wide association study (GWAS) [13].

Environmental risk factors for CAKUT have also been studied for many years, with an increasing focus on differences between CAKUT phenotypes [14, 15]. Among the risk factors implicated in the aetiology of CSFK are conception using *in vitro* fertilization/intracytoplasmic sperm injection and maternal diabetes, obesity, smoking, infections and stress [15–19]. Genetic and environmental factors are, however, insufficient to explain the aetiology of many congenital anomalies, including CSFK. Part of this missing insight into the aetiology may be explained by gene–environment (G×E) interactions [20]. Although evidence for interactions between genetic variants and environmental factors is available for other congenital anomalies [21–23], no studies on G×E interactions have yet been performed for CSFK. Therefore, we combined data on common genetic variants and exposure to environmental factors to explore the role of G×E interactions in the aetiology of CSFK.

## MATERIALS AND METHODS

### Study participants

Study participants were derived from the AGORA (Aetiologic research into Genetic and Occupational/environmental Risk factors for Anomalies in children) data- and biobank [24], and were included in a previous GWAS [13] and a study on environmental risk factors [17]. Briefly, parents of patients with CSFK, defined as a solitary functioning kidney resulting from unilateral kidney agenesis, MCDK or hypo-/dysplasia, were asked to participate in AGORA when visiting the Radboud University Medical Center (from 2004 onwards) or University Medical Center Utrecht (from 2013 onwards). Additional recruitment to increase the number of CSFK patients in AGORA was initiated in 2018, when CSFK patients from 36 hospitals throughout the Netherlands were enrolled in the SOFIA study [1]. Biological parents were asked to fill out a questionnaire on environmental exposures shortly before and during the pregnancy of their child. DNA of the patients was extracted from blood (when available) or saliva. Patients with a known or suspected genetic or syndromic aetiology were excluded from the current study. Healthy controls were recruited through a random sample of children living in 39 collaborating Dutch municipalities. All parents of these children were also asked to fill out the questionnaire and some of them to provide a saliva sample from their child. Only children without major birth defects (defined using EUROCAT guidelines [25]) were eligible as controls. The AGORA data- and biobank was approved by the Regional Committee on Research Involving Human Subjects.

### Environmental risk factors

A full description of the assessment of environmental exposures was published previously [17]. From this larger set of potential risk factors, six were selected as most likely to interact with genetic variants by a panel of experts in reproductive and genetic epidemiology: parental subfertility, use of assisted reproductive techniques (ART), maternal overweight/obesity, maternal folic acid supplementation, maternal smoking and maternal alcohol consumption during the aetiologically relevant time period. To study parental subfertility, couples reporting a diagnosis of subfertility, defined as a time to pregnancy of >12 months, or conception using ART, were compared with parents who conceived naturally within 12 months. To study ART specifically, we compared the subgroup of patients who conceived using ART with the same control group, excluding parents reporting subfertility but no use of ART from the analysis. Maternal overweight/obesity was defined as a body mass index (BMI) >25 kg/m^2^ at the time of conception. Folic acid supplementation was considered as use of folic acid supplements or folic acid–containing multivitamins during the entire advised period (initiation before pregnancy and continued through at least the eighth week of pregnancy). Maternal smoking and alcohol use were both defined as exposure in the 3 months before and/or during pregnancy.

### Genotyping, quality control, and imputation

The genetic data and procedures on quality control and imputation were derived from a previous GWAS into the aetiology of CSFK [13]. Genotyping was performed by deCODE genetics (Reykjavik, Iceland) using Infinium Global Screening Arrays (Illumina, San Diego, CA, USA) in separate batches for patients and controls. During quality control, variants with a call rate <98%, a deviation from Hardy–Weinberg equilibrium with a *P*-value <1 × 10^−10^ (for patients) or <1 × 10^−6^ (for controls), or a minor allele frequency <0.01 were removed. In addition, samples were excluded if they had a call rate <98% or if individuals were related up to the third degree or were of non-European ancestry (ancestries other than 1000 Genomes population codes GBR or CEU) [26]. Imputation was performed for patients and controls together based on the overlap in variants genotyped (*n* = 524 412) using Minimac (v4), with 1000 Genomes Project Phase 3 data as reference panel.

### Statistical analyses

We applied the two-step analytical strategy proposed by Murcray *et al*. [27], in which the *P*-value threshold in the main G×E interaction analysis (step two) is based on a weighting step (step one). This method was shown to be more powerful compared with a one-step strategy when the exposure or the disease allele are rare [27]. In step one, we estimated the associations between all single-nucleotide variants (SNVs) and the selected environmental factors among the combined sample of patients and controls. We used FUMA to select independent (r^2^ ≤ 0.6) SNVs with a *P*-value ≤1 × 10^−5^ for the association with exposure status [28]. For the each of the selected SNVs, we fitted logistic regression models for case–control status in step two, with the following terms: exposure main effect, SNV main effect, and the exposure × SNV interaction term. We selected the first four principle components, maternal level of education and sex of the child as potential confounders. To properly adjust for confounding [29], covariate main effects, covariate × environment interaction terms and covariate × gene interaction terms were added to the models. For the analyses in step two, the *P*-value threshold for statistical significance was determined as .05 divided by the number of SNVs selected in step one for the exposure of interest, but SNVs with a *P*-value <.05 were also selected as suggestive loci for further inspection. The selected loci were visualized using LocusZoom [30] and assessed for functional relevance using the RegulomeDB [31]. Lastly, expression quantitative trait loci (eQTLs) were determined using the Genotype-Tissue Expression (GTEx) portal (https://gtexportal.org/). Genetic quality control and the analyses in step one were performed with PLINK 2.0 [32], while the analyses in step two were performed in R version 4.1.0 [33].

## RESULTS

**Figure 1:**
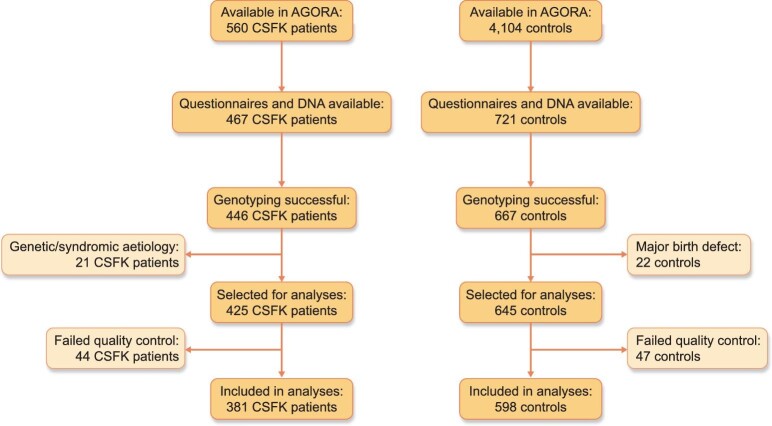
Flowchart of inclusion of patients and controls.

In total, 560 CSFK patients and 4104 controls were available in the AGORA data- and biobank. Questionnaires were available for 501 patients and 4039 controls, whereas DNA was collected from 503 patients and 725 controls. From 467 patients and 721 controls, both questionnaires and DNA were present. Genotyping was successful in 446 of these patients and 667 controls, but 21 patients were excluded because of a known or suspected genetic or syndromic aetiology and 22 controls because of a major birth defect. After genomic quality control and imputation, information on 9 956 431 SNVs from 381 patients and 598 controls was available for use in the G×E interaction analyses. A flowchart of the inclusion is presented in Fig. 1. Patients were more often male and their mothers were more often highly educated compared with controls (Table 1). Patients were also more likely to be conceived using ART and to have a mother who was overweight or obese, whereas maternal age and parity as well as the frequency of the other exposures were comparable between patients and controls.

**Table 1:** Overview of maternal and pregnancy characteristics among patients with CSFK as well as population-based control subjects.

	Patients,	Controls,
	*n* = 381 (%)	*n* = 598 (%)
Maternal age group		
≤25 years	20 (5)	22 (4)
25–34.9 years	275 (72)	468 (78)
≥35 years	77 (20)	108 (19)
Missing	9 (2)	0 (0)
Parity		
Nulliparous	156 (41)	232 (39)
Multiparous	210 (55)	364 (61)
Missing	15 (4)	2 (0)
Child sex		
Male	245 (64)	291 (49)
Female	136 (36)	307 (51)
Maternal education level		
Low	45 (12)	94 (16)
Intermediate	149 (39)	289 (48)
High	187 (49)	214 (36)
Missing	0 (0)	1 (0)
Parental subfertility		
No subfertility	293 (77)	458 (77)
Any subfertility	84 (22)	88 (15)
Missing	4 (1)	52 (9)
Conception using ART		
No subfertility	293 (77)	458 (77)
Conception using any ART	34 (9)	17 (3)
Missing	54 (14)	123 (21)
Maternal BMI		
≤25 kg/m^2^	252 (66)	431 (72)
>25 kg/m^2^	119 (31)	139 (23)
Missing	10 (3)	28 (5)
Maternal folic acid supplementation		
As recommended	173 (45)	245 (41)
Other	187 (49)	317 (53)
Missing	21 (6)	36 (6)
Maternal smoking		
No	308 (81)	470 (79)
Yes	72 (19)	128 (21)
Missing	1 (0)	0 (0)
Maternal alcohol use		
No	218 (57)	319 (53)
Yes	160 (42)	279 (47)
Missing	3 (1)	0 (0)

In step one, the number of independent SNVs with a *P*-value ≤1 × 10^−5^ varied per environmental factor between 7 (maternal folic acid supplementation) and 40 (conception through ART) (Table 2). The lowest *P*-values for associations with exposure to the environmental factors ranged between 1.4 × 10^−6^ (maternal alcohol use) and 1.0 × 10^−8^ (maternal smoking). All SNVs selected in step one were carried forward to the G×E interaction analysis in step two. Of these SNVs, one reached the Bonferroni-corrected threshold for statistical significance (rs3098698, *P*-value .0016, threshold 0.0045; Minimac imputation quality score 0.96785). This variant is an intronic variant of the Arylsulfatase B (*ARSB*) gene, with a RegulomeDB score of 0.13 and an odds ratio (OR) for the corresponding G×E interaction of 3.2 [95% confidence interval (CI) 1.5–7.2] (Table 3). ORs for CSFK comparing mothers with overweight/obesity to normal-weighted mothers stratified per genotype of the child were 1.9 (95% CI 1.3–2.6) in children homozygous for the reference allele and 0.6 (95% CI 0.3–1.3) for heterozygous children (Table 4). Too few children (two patients and five controls) carried two alternative alleles to calculate the OR for this group. Eight other SNVs reached a *P*-value <.05 (Table 3), of which two are located in regulatory regions of CTCF (CCCTC-Binding Factor) binding sites, five are located in an intron and one is located between genes. RegulomeDB scores varied between 0.01 and 0.80, reflecting a very low and high probability of being a regulatory variant, respectively [34]. The point estimates for the OR of G×E interaction varied between 0.1 and 51.4. For only two variants (rs13409540 and rs5796570), ORs for the corresponding environmental factor (maternal overweight/obesity and folic acid supplementation, respectively) could be assessed in all three genotypes, with a dose–response visible for rs5796570 but not for rs13409540 (Table 4).

**Table 2:** Number of independent SNVs associated with each environmental risk factor in complete case analyses.

	Number of independent significant SNVs^a^	Lowest *P*-value in step one	Bonferroni-corrected *P*-value threshold in step two
Any subfertility	12	9.1 × 10^−8^	.0042
Conception using ART	40	1.3 × 10^−7^	.0013
Maternal BMI	11	7.3 × 10^−7^	.0045
Maternal folic acid supplementation	7	1.4 × 10^−7^	.0071
Maternal smoking	20	1.0 × 10^−8^	.0025
Maternal alcohol use	10	1.4 × 10^−6^	.0050

a Independent significant SNVs were those with an r^2^ ≤ 0.6 and a *P*-value ≤1 × 10^−5^ for the association with the environmental risk factor of interest.

**Table 3:** Location and predicted effect of selected single nucleotides with a gene–environment interaction *P*-value <.05.

Environmental factor	Chr	Position^a^	Ref	Alt	rs number	OR G×E (95% CI)	*P*-value G×E	Variant effect predictor	Gene	RegulomeDB score^b^
ART	Chr 4	99 731 659	C	A	rs75166568	0.1 (0.0–0.4)	.0055	Intergenic		0.22
ART	Chr 4	99 749 971	A	G	rs35047415	0.1 (0.0–0.6)	.0087	Regulatory region variant	*CTCF* binding site	0.57
ART	Chr 13	26 755 829	T	C	rs77612507	51.4 (1.4–501)	.0298	Intronic variant	*RNF6*	0.32
BMI	Chr 2	67 196 296	A	G	rs13409540	0.5 (0.3–0.8)	.0057	Intronic variant	*ETAA1*	0.61
**BMI**	**Chr 5**	**78 174 969**	**G**	**A**	**rs3098698**	3.2 (1.5–7.2)	**.0016**	**Intronic variant**	***ARSB***	**0.13**
BMI	Chr 15	52 266 922	T	C	rs367873445	2.6 (1.2–5.8)	.0177	Intronic variant	*MAPK6*	0.01
Smoking	Chr 1	37 900 415	C	T	rs11264047	0.4 (0.2–0.9)	.0208	Regulatory region variant	*CTCF* binding site	0.80
Smoking	Chr 7	8 559 913	T	A	rs75063030	5.2 (1.4–22.3)	.0125	Intronic variant	*NXPH1*	0.50
Folic acid	Chr 12	14 123 620	A	AAC	rs5796570	0.6 (0.4–0.9)	.0251	Intronic variant	*GRIN2B*	0.13

Bold values indicate associations with a *P*-value below the Bonferroni-corrected threshold for statistical significance.

a Genomic locations are represented in genome build GRCh37/Hg19.

b The RegulomeDB score ranges from 0 to 1 and represents the likelihood of being a regulatory variant [34].

Chr, chromosome; Ref, reference allele; Alt, alternative allele.

**Table 4:** ORs with 95% CIs for the association between environmental factors and CSFK stratified per SNV genotype.

		Reference genotype			Heterozygous genotype			Variant genotype		
Environmental factor	rs number	*N* (%) patients	*N* (%) controls	OR (95% CI)	*N* (%) patients	*N* (%) controls	OR (95% CI)	*N* (%) patients	*N* (%) controls	OR (95% CI)
ART	rs75166568	295 (77)	457 (76)	1.9 (0.8–4.5)	81 (21)	133 (22)	6.2 (2.3–17)	2 (1)	7 (1)	n/a
ART	rs35047415	314 (82)	473 (79)	2.3 (1.1–5.3)	64 (17)	117 (20)	6.7 (2.3–19)	2 (1)	6 (1)	n/a
ART	rs77612507	367 (96)	584 (98)	4.2 (2.1–8.4)	13 (3)	14 (2)	0.8 (0.1–4.8)	0	0	n/a
BMI	rs13409540	221 (58)	333 (56)	1.1 (0.7–1.6)	137 (36)	221 (37)	2.5 (1.6–4.1)	23 (6)	44 (7)	2.0 (0.6–6.5)
BMI	rs3098698	312 (82)	509 (85)	1.9 (1.3–2.6)	67 (18)	84 (14)	0.6 (0.3–1.3)	2 (1)	5 (1)	n/a
BMI	rs367873445	317 (83)	527 (88)	1.7 (1.3–2.4)	62 (16)	70 (12)	0.7 (0.4–1.5)	2 (1)	1 (0)	n/a
Smoking	rs11264047	322 (85)	497 (83)	0.8 (0.5–1.1)	57 (15)	94 (16)	2.8 (1.3–5.9)	2 (1)	7 (1)	n/a
Smoking	rs75063030	351 (92)	561 (94)	1.1 (0.8–1.6)	28 (7)	35 (6)	0.3 (0.1–0.9)	2 (1)	2 (0)	n/a
Folic acid	rs5796570	161 (42)	270 (45)	0.6 (0.4–0.9)	177 (47)	275 (46)	1.0 (0.7–1.6)	43 (11)	50 (8)	1.4 (0.6–3.5)

n/a, not applicable.

## DISCUSSION

Our study is the first genome-wide G×E interaction study in patients with CSFK and identified one statistically significant interaction between the genetic variant rs3098698 and the environmental factor maternal overweight/obesity. Eight other interactions reached a *P*-value <.05, but were above the Bonferroni corrected *P*-value threshold. Despite the small sample size, this study illustrates the potential value of gene–environment interaction studies in the field of CAKUT.

**Figure 2:**
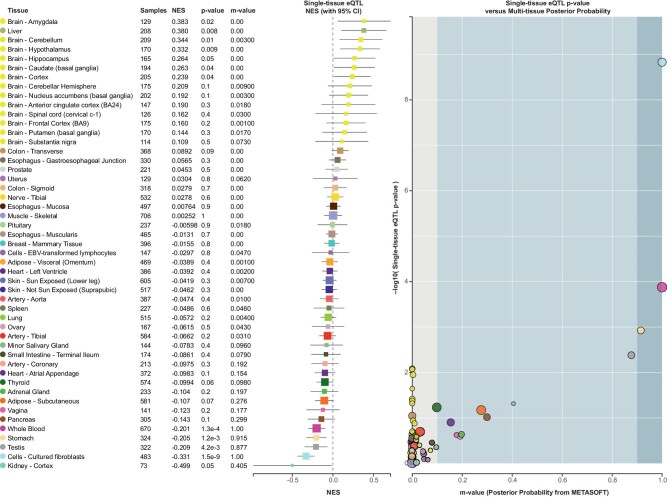
Single-tissue eQTL normalized expression scores of ARSB for rs3098698 in different cell types. Figure created using GTEx portal (https://gtexportal.org/).

To explain the statistically significant interaction between variant rs3098698 on chromosome 5 and maternal overweight/obesity, functional consequences of this SNV were studied. The variant is located in an intron of the *ARSB* gene (Supplementary data, Fig. S1), a gene that codes for a sulfatase (Arylsulfatase B). Arylsulfatase B controls degradation of chondroiting4-sulfate and dermatan sulphate, which may in turn influence intracellular signalling, cell–cell communication and transcription [35]. When expression of the *ARSB* gene is reduced, chondroiting4-sulfate is more abundant, which leads to increased cellular and nuclear galectin-3 levels [36]. Galectin-3 binds to the insulin receptor and causes insulin resistance [37]. The GTEx Portal indicates that the rs3098698 variant is associated with lower expression of *ARSB*, with the largest estimated effect size in kidney cortex cells (Fig. 2). Therefore, children heterozygous for the rs3098698 variant are expected to be more resistant to insulin. Maternal overweight/obesity did not increase the risk of CSFK in these children, possibly because the increased maternal glucose levels do not result in increased cellular uptake of glucose in children with the SNV because of their lower insulin receptor activity. Experimental work should further investigate this and other potentially relevant mechanisms involved and could provide insight into whether the rs3098698 variant is the causal SNV or acts as proxy for other variants that were not directly tested. For example, the rs163215 variant, which is in linkage disequilibrium with rs3098698, is more likely to have functional effects based on RegulomeDB scores and eQTL data. Fine mapping of this region is needed to get more insight into which variant is causal.

Eight other G×E interactions reached a *P*-value <.05. Three of the variants involved (rs35047415 on chromosome 4 interacting with ART, rs13409540 on chromosome 2 interacting with maternal BMI and rs11264047 on chromosome 1 interacting with maternal smoking) showed RegulomeDB scores above 0.5. One other variant (rs367873445 on chromosome 15, which showed an interaction with BMI) was a statistically significant eQTL. Therefore, these four variants are most likely to have functional consequences as well.

The highest RegulomeDB score was observed for rs11264047, a variant that showed an interaction with maternal smoking and results in higher expression of LINC01137 in several tissues, including kidney cortex tissue. Similar to smoking [38], LINC01137 inhibits formation of miR125-b [39], thereby possibly influencing the balance between transforming growth factor beta and Wnt signalling, which are important signalling pathways during kidney development [40–42]. Variant rs35047415 on chromosome 4 was predicted as regulatory variant with an eQTL indicating slightly increased expression of *ADH5* in several tissues but not in kidney tissue. The encoded ADH5 protein is involved in aldehyde clearance, which can be linked to congenital anomalies [43], but not to use of ART. The third variant with a high RegulomeDB score was rs13409540 on chromosome 2, which showed an interaction with maternal BMI. This variant is located in an intron of *ETAA1*, a gene involved in maintaining genomic stability [44]. No effect on gene expression was found based on eQTL data however, making functional consequences less likely. Lastly, rs367873445 on chromosome 15 showed an interaction with maternal BMI as well. The GTEx Portal indicates that this variant may lead to higher expression of *SCG3* and *MYO5C* in kidney tissue, which have been linked to insulin secretion and overweight, respectively [45, 46]. The interaction between rs367873445 on chromosome 15 and BMI, as well as the interaction between rs11264047 on chromosome 1 and maternal smoking, are promising to investigate in future studies.

Our study used data from a previous GWAS and a previous study on environmental risk factors for CSFK [13, 17]. As such, the limitations of these studies are also relevant for the current G×E interaction study. Most importantly, the number of patients, although large for a study into a specific type of congenital anomaly, limits the power to detect associations. Power calculations using Quanto [47] indicated a power of approximately 80% to detect a G×E interaction, assuming a disease prevalence of 1:1500, an SNV with a log-additive mode of inheritance, a minor allele frequency of 0.05 with an OR of 3.0, and an exposure with a prevalence of 20% and an OR of 1.5. Power to detect an interaction with an OR of 2.0 was estimated to be only 40%, although the two-step approach likely increased our power substantially [27]. Nevertheless, the limited number of subjects prevented us from further stratification into specific causes of CSFK (i.e. agenesis, MCDK and hypo-/dysplasia), which may be relevant because of potential differences in aetiology. Furthermore, participants were not systematically screened for monogenic causes of CSFK or pathogenic CNVs, although patients with known or suspected genetic or syndromal causes were excluded. All environmental factors were assessed using self-reported questionnaires completed after delivery. This could have introduced the risk of recall bias for some lifestyle factors, although the period around pregnancy may be expected to be well-remembered by mothers. Similar to SNVs detected in a regular GWAS, the SNVs identified in our study may act as proxies for other causal but untested genetic variants. We tried to establish the effects of variants using *in silico* analyses, which clearly have their limitations. For example, the eQTL data used were derived from adult tissue, which may not be representative for the aetiologically relevant time period. The main strength of this study consists of the well-defined population in which a combination of data on genetic and environmental risk factors was available. In addition, a genome-wide search was possible due to the efficient design of the study, maximizing power in the available population.

The current study explored the possibility of G×E interactions in the aetiology of CSFK and found indications for several such interactions. Limited statistical power and unavailability of replication cohorts mitigate the current implications of our results, which warrant confirmation before firm conclusions can be drawn. The study illustrates, however, that the aetiology of CSFK is complex. Integration of clinical data, genetic data and information on environmental risk factors is needed for an integral assessment of relevant aetiological mechanisms, while large numbers of patients are needed to facilitate adequately powered studies. Future studies should aim for collection of all of these types of data to avoid research being confined to only one aspect of the aetiology of CSFK.

In conclusion, we identified G×E interactions as a relevant aetiological mechanism for CSFK, warranting further studies on this topic. We found an important interaction between the rs3098698 variant and the effect of maternal overweight/obesity, which provides novel leads for the pathophysiology of CSFK. This and several other potentially relevant combinations of genetic variants and environmental factors may contribute to CSFK development, although replication of our findings is highly warranted. Future studies should focus on genetic and environmental factors, as well as their interaction, to allow for a comprehensive view on the aetiology of CSFK.

## Supplementary Material

gfad202_Supplemental_File

## Data Availability

Data are available from the authors upon reasonable request.
