# Supplementary figures and images for: Genetic and environmental factors driving congenital solitary functioning kidney
Source: Nephrol Dial Transplant. 2023 Sep 20;39(3):463–72. doi: 10.1093/ndt/gfad202 (PMC10899751; doi:10.1093/ndt/gfad202)

## Slide 1
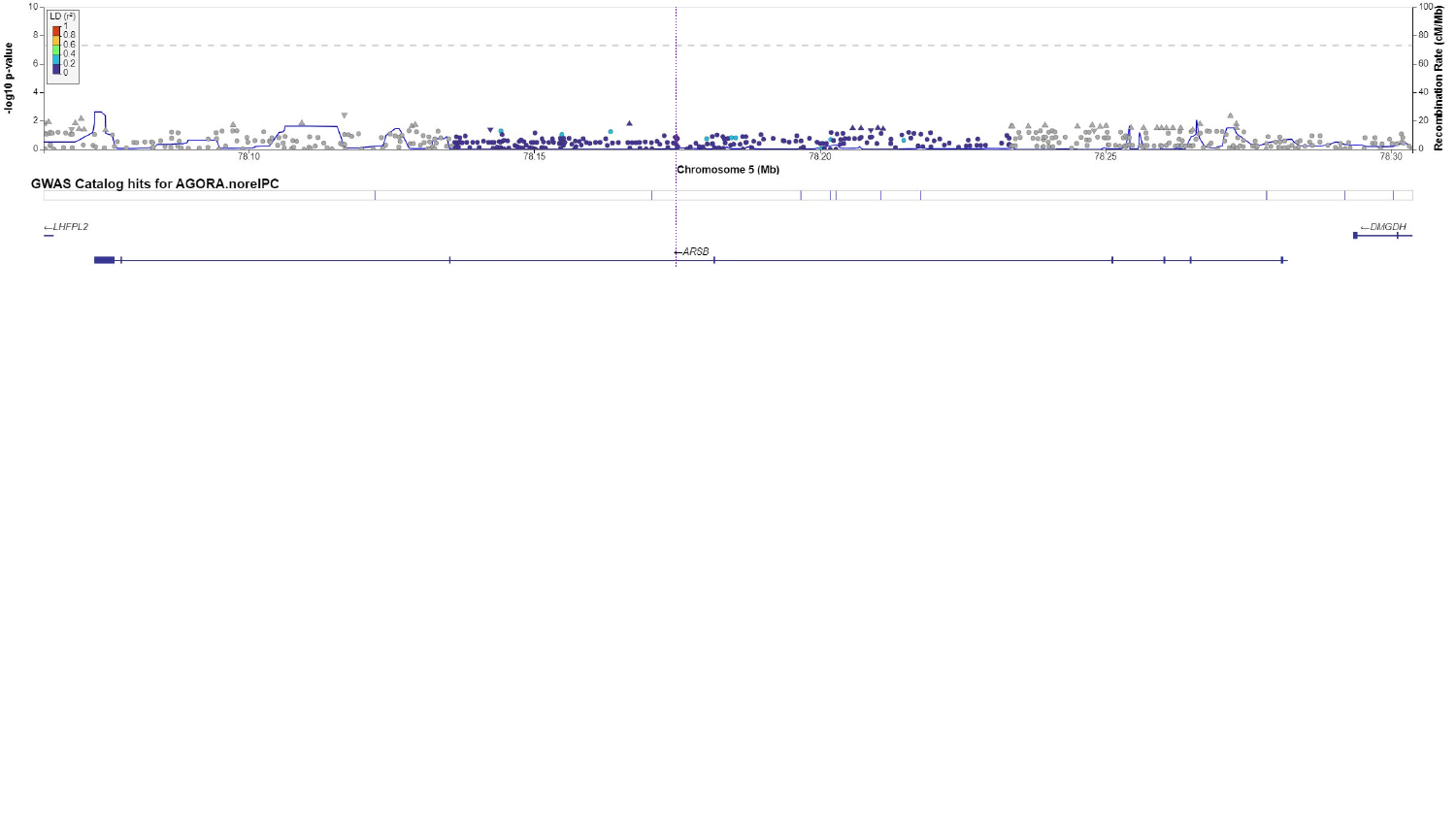

Supplement: gfad202_Supplemental_File [file gfad202_supplemental_file.pptx]
